# Xrn1 is a deNADding enzyme modulating mitochondrial NAD-capped RNA

**DOI:** 10.1038/s41467-022-28555-7

**Published:** 2022-02-16

**Authors:** Sunny Sharma, Jun Yang, Ewa Grudzien-Nogalska, Jessica Shivas, Kelvin Y. Kwan, Megerditch Kiledjian

**Affiliations:** Department of Cell Biology and Neuroscience, Rutgers University, Piscataway, NJ 08854 USA

**Keywords:** Biochemistry, RNA metabolism

## Abstract

The existence of non-canonical nicotinamide adenine diphosphate (NAD) 5′-end capped RNAs is now well established. Nevertheless, the biological function of this nucleotide metabolite cap remains elusive. Here, we show that the yeast *Saccharomyces cerevisiae* cytoplasmic 5′-end exoribonuclease Xrn1 is also a NAD cap decapping (deNADding) enzyme that releases intact NAD and subsequently degrades the RNA. The significance of Xrn1 deNADding is evident in a deNADding deficient Xrn1 mutant that predominantly still retains its 5′-monophosphate exonuclease activity. This mutant reveals Xrn1 deNADding is necessary for normal growth on non-fermenting sugar and is involved in modulating mitochondrial NAD-capped RNA levels and may influence intramitochondrial NAD levels. Our findings uncover a contribution of mitochondrial NAD-capped RNAs in overall NAD regulation with the deNADding activity of Xrn1 fulfilling a central role.

## Abstract (web summary)

The cytoplasmic Xrn1 protein has long been established as the predominate 5′ to 3′ exoribonuclease that cleaves RNAs with an unprotected 5′ monophosphate end. Here the authors demonstrate Xrn1 can also degrade RNAs harboring the noncanonical nicotinamide adenine diphosphate (NAD) 5′ cap by removing the NAD cap and degrading the RNA.

## Introduction

Nucleotide metabolites serve as cofactors for multiple enzymatic activities essential for cellular survival and propagation^1^. Recent demonstrations of the redox cofactor NAD covalently linked to the 5′-end of RNAs as an RNA cap in bacteria, yeast, plants, and mammals have uncovered a modulatory network where a metabolite can directly impact gene expression^2–5^. Biochemical studies in prokaryotes have shown that bacterial polymerases can add an NAD cap in place of ATP at the 5′-end during transcription initiation^6^. On the other hand, detection of an NAD cap on post-transcriptionally processed intronic small nucleolar RNAs (snoRNAs) in mammalian cells has advocated the existence of a post-transcriptional NAD-capping mechanism as well^3^. These studies have also led to the identification of a battery of NAD-cap decapping (deNADding) enzymes—NudC in bacteria^2,7,8^ and its human homolog Nudt12^9,10^, Nudt16^10^ and the DXO/Rai1 family of non-canonical decapping enzymes^3,11^. DXO/Rai1 are class 1 deNADding enzymes that target the phosphodiester linkage and releases an intact NAD while NudC, Nudt12, and Nud16 are class 2 deNADding enzymes that hydrolyze the pyrophosphate bond within the NAD of the NAD cap and release nicotinamide mononucleotide (NMN)^9,12^.

Functional analyses of the NAD-capped RNAs have revealed disparate roles in bacterial and mammalian cells. In bacteria, the NAD cap is considered to protect the RNA from 5′-end degradation^2^, whereas in mammals^3^ and plants^13^ NAD caps have been shown to promote RNA decay. In bacteria, NAD caps are found on certain regulatory RNAs^2^, while in budding yeast *Saccharomyces cerevisiae*, they are on a subset of mitochondrial RNAs and transcripts encoding the translational machinery^4^. NAD-capped RNAs are prevalent in the transcriptome of plants^5^ and human cells^3^, and are mainly comprised of mRNAs and small nucleolar RNAs (snoRNAs). The NAD cap does not appear to support translation in mammalian cells^3^, while their presence on plant polysomes indicates a translational capacity^5^. Despite the occurrence of NAD caps in diverse organisms, the biological role of this non-canonical metabolite cap in cellular physiology has remained elusive.

## Results

### Identification of Xrn1 and Rat1 as NAD cap associated proteins

**Fig. 1:**
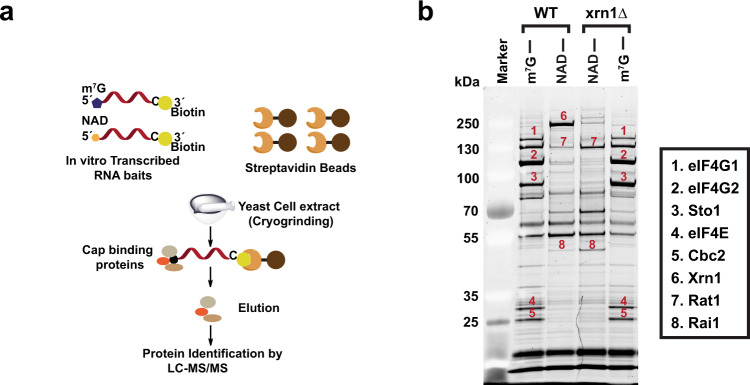
Identification of NAD cap-binding proteins in budding yeast. **a** Schematic illustration of the NAD cap -RNA Affinity Purification (NcRAP). **b** Eluates from NcRAP were loaded on to a 4–12% Bis–Tris gel and stained with SYPRO Ruby. The m^7^G cap affinity purification was used as a control. All protein bands labeled on the gel were excised from the gel and identified using mass spectrometry.

To begin unraveling the functional role of NAD caps, we set out to identify proteins that specifically bind to the NAD cap. Protein purification was carried out by 5′**N**AD **c**ap **R**NA **A**ffinity **P**urification (NcRAP) with the RNA containing a 3′-biotin to immobilize the affinity matrix and identify proteins associated with the NAD cap from *S. cerevisiae* lysate as illustrated in Fig. 1a. Canonical m^7^G-capped RNA was used as a control for the cap affinity purification and associated proteins were identified by mass spectrometry. As expected, the nuclear (Sto1 and CBC2) and cytoplasmic (eIF4E) canonical cap-binding proteins were prominent bands selectively detected on the m^7^G cap RNA affinity column as well as the associated eIF4F adapter complex proteins eIF4G1 and eIF4G2^14^ (Fig. 1b and Supplementary Fig. 1a), demonstrating the feasibility of the NcRAP approach.

Mass spectrometry identification of proteins selectively bound to the NAD-capped RNA (Supplementary Data 1) revealed surprising candidates. The most prominent protein interacting with the NAD cap was the cytoplasmic 5′-3′ exoribonucleases Xrn1^15^ (Fig. 1b). Additional prominent bands unique to the NAD cap lane included the nuclear 5′-3′ exoribonucleases Rat1 (Xrn2 in mammals)^16,17^ and the Rat1 interacting partner, Rai1^18^ (Fig. 1b and Supplementary Fig. 1a). These interactions were further validated by performing NcRAP using protein lysates derived from an Xrn1 deletion (*xrn1∆)* strain. In the absence of Xrn1, binding of both the Rat1 and Rai1 proteins to the NAD cap was accentuated. Moreover, since Rat1 and Rai1 exist as heterodimers^18^ and Rai1 is a known deNADding protein^3^, extract from the *rai1Δ* strain was used in NcRAP. As shown in Supplementary Fig. 1b, the association of Rat1 with the NAD cap was independent of its interaction with the Rai1 based on its retention onto the NAD-capped RNA column. These findings reveal Xrn1, Rat1 and Rai1 are capable of selectively binding to the NAD cap under the assay conditions employed.

### Xrn1 and Rat1 hydrolyze NAD-capped RNAs in vitro

**Fig. 2:**
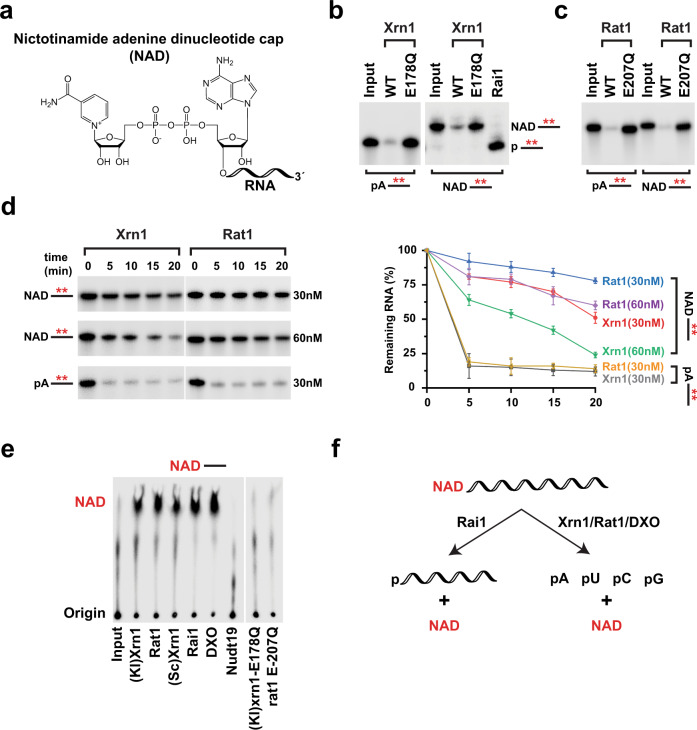
Xrn1 and Rat1 are deNADding enzymes. **a** Structure of NAD-capped RNA. **b** Reaction products of in vitro deNADding assays with 30 nM recombinant Xrn1, WT, and catalytically inactive (*E178Q*) from *K. lactis or* 25 nM Rai1 (*S. pombe)*, (**c**) and 50 nM recombinant Rat1, WT or catalytically inactive (*E207Q*) from *S. pombe*. Uniformly ^32^P-labeled monophosphate (pA----) or NAD-capped RNA (NAD---) were used as indicated and the line denotes the RNA. The red asterisk represents the ^32^P-labeling within the body of the RNA. Reaction products were resolved on 15% 7 M urea PAGE gels with (**b**) and without (**c**) 0.3% 3-acrylamidophenylboronic acid. **d** Time-course decay analysis of uniformly ^32^P-labeled monophosphate or NAD-capped RNA (9pmol) with the indicated amount of Xrn1 or Rat1 (~0.45 or ~0.9 pmol, respectively) protein are shown. Quantitation of RNA remaining is plotted from *n* = 3 independent experiments with ±SD denoted by error bars. Source data are provided in Source Data File. **e** NAD-capped RNA containing the ^32^P-labeling within the α phosphate of the NAD (represented in red) was subjected to the indicated proteins. Decay products were resolved by polyethyleneimine (PEI)-cellulose thin layer chromatography (TLC) developed in 0.45 M (NH_4_)_2_SO_4_. Rai1 and DXO served as positive controls, whereas Nudt19 was a negative control. **f** Schematic of NAD-capped RNA hydrolysis by Rai1, Xrn1, Rat1 and DXO.

Identification of Xrn1 and Rat1 by NcRAP in addition to Rai1, a potent deNADding enzyme^3^, raised the intriguing possibility that analogous to Rai1, Xrn1 and Rat1 may also possess deNADding activity. Since both Xrn1 and Rat1 are highly processive 5′-monophosphate (5′P)-specific 5′-3′ exoribonucleases^15,17^, we assessed the potential of these enzymes to hydrolyze 5′NAD-capped RNA (Fig. 2a) in vitro. As expected, Xrn1 efficiently degraded 5′P RNA (Fig. 2b) but not a 5′-triphosphate- or an m^7^G-capped RNA (Supplementary Fig. 2a). Importantly, uniformly ^32^P-labeled NAD-capped RNA was degraded by wild-type *Kluyveromyces lactis* (Fig. 2b) and *S.cerevisiae* (Fig. 2b) Xrn1, but not the catalytically inactive *xrn1-E178Q*^19^ mutant protein. Interestingly, the decay activity of Xrn1 on NAD-capped RNA was processive without detectable intermediates in contrast to *Schizosaccharomyces pombe* Rai1, which removed the NAD cap without subsequent degradation of the deNADded RNA (Fig. 2b), consistent with our previous report^3^. Similarly, *S.pombe* Rat1 also hydrolyzed the NAD-capped RNA with processive exonuclease activity, whereas the catalytically dead *rat1-E207Q*^20^ was inactive (Fig. 2c). Collectively, these data demonstrate that the observed degradation of the NAD-capped RNA is a function of Xrn1 and Rat1, and requires the same catalytic active site utilized for the hydrolysis of a 5′P RNA.

To further understand the kinetics of deNADding, a time-course decay assay was carried out with 5′P- or NAD-capped RNA using two different concentrations of Xrn1 and Rat1. Consistent with the well characterized exoribonuclease activities of Xrn1 and Rat1, both effectively degraded 5′P RNA (Fig. 2d). Importantly, the activity of Xrn1 on 5′P RNA was 20 fold more efficient than NAD-capped RNA at comparable enzyme concentrations (Fig. 2d and Supplementary Fig. 2d). Furthermore, Xrn1 degraded the NAD-capped RNA twice as efficiently as Rat1. To assess whether Xrn1 hydrolyzed the NAD cap within the diphosphate moiety analogous to the class 2 Nudt12 protein, or removed the intact NAD, similar to the class 1 DXO/Rai1 family of proteins^12^, thin layer chromatography (TLC) was used. Interestingly, ^32^P-labeled NAD-capped RNA subjected to Xrn1 or Rat1 hydrolysis released intact NAD (Fig. 2e). In addition, unlike Nudt12 both Xrn1 and Rat1 do not hydrolyze the diphosphate bond of free NAD (Supplementary Fig. 2e). We conclude that Xrn1 and Rat1 are class 1 deNADding enzymes that have the capacity to degrade NAD-capped RNA analogous to the DXO/Rai1 family of proteins by removing the intact NAD (Fig. 2f). These findings increase the repertoire of substrate RNAs regulated by Xrn1 and Rat1 beyond their well characterized 5′-monophosphate directed exoribonuclease activity. Moreover, the deNADding activity of Xrn1 is not restricted to yeast, as shown in Supplementary Fig. 2f, human Xrn1 also hydrolyzes NAD-capped RNAs.

### Loss of Xrn1 stabilizes NAD-capped RNAs in vitro and in vivo

**Fig. 3:**
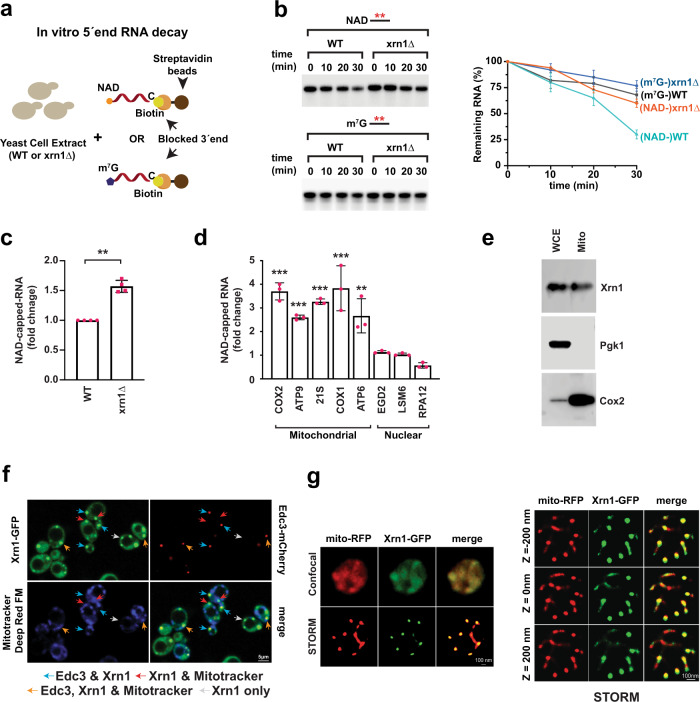
Xrn1 deNADs NAD-capped RNAs in cells. **a** Schematic illustration of in vitro 5′-end RNA decay. **b** Time-course decay analysis of uniformly ^32^P-labeled m^7^G- or NAD-capped RNA in the presence of cell extract prepared from WT or *xrn1Δ* strains. The remaining RNA was quantified and plotted from *n* = 3 independent experiments (±SD denoted by error bars). Labeling as in the legend to Fig. 2. **c** Total NAD-capped RNA levels from WT and *xrn1Δ* were detected by NAD-capQ. Data represents average from *n* = 4 independent experiments. Error bars represent ±SEM; two sided unpaired *t* test; **p = 0.0036). **d** qRT-PCR quantification of NAD-capped mRNA. NAD-capped RNA was enriched by NAD-capture, eluted from the beads, reverse transcribed and detected with gene-specific primers. Data are presented relative to the WT cells set to 1 and derived from *n* = 3 independent NAD-capture experiments; mean ± SEM; two-sided unpaired *t* test; **p < 0.001, ***p < 0.0001. **e** Purified mitochondria derived from a strain harboring Xrn1 with a C-terminus Strep-tag II was analyzed by Western blot analysis with an anti-Strep-tag antibody. Western blotting analysis of whole cell extract (WCE) and extract prepared from purified mitochondria (mito) using Pgk1 (cytoplasmic protein) and Cox2 (mitochondrial protein) to determine the purity of mitochondria. Source data for panels b, c and d are provided in Source Data File**. f** Xrn1 knock-in strain containing the gene encoding green fluorescence protein (GFP) in frame with endogenous Xrn1 was generated into the strain carrying Edc3 fused to mCherry. Colocalization of Xrn1 with mitochondria (Mitotracker® Deep Red FM) and Edc3 (mCherry) by standard confocal (40X) microscopy is shown. **g** Reconstructed STORM images. Left panel shows images from a single optical section of RFP-labeled mitochondria and GFP-labeled Xrn1 in a single yeast cell obtained by spinning disk confocal (top) and STORM imaging (bottom). Merged image shows overlap of RFP and GFP signal. Right panel shows reconstructed STORM images obtained from sequentially acquired optical sections spaced 200 nm apart in the z axis. Merged images show colocalization of signals within the ~10 nm XY resolution. Scale bar is 100 nm.

Having demonstrated Xrn1 and Rat1 function as deNADding enzymes in vitro, we assessed the deNADding activity of Xrn1 in cells. We focused on Xrn1 since the *xrn1Δ* strain is viable and amenable to genetic manipulation, while the *rat1Δ* stain is not^16^. 5′-end RNA decay assays were carried out with extract derived from either wild-type (WT) or *xrn1∆* cells using NAD-or m^7^G-capped RNAs immobilized and protected at the 3′-end with biotin-streptavidin (Fig. 3a). While no significant differences were observed with the control m^7^G-capped RNA, the NAD-capped RNA was more stable in the *xrn1Δ* extract, suggesting endogenous Xrn1 possesses deNADding activity (Fig. 3b). To assess the contribution of Xrn1 deNADding on endogenous NAD-capped RNA, we used the NAD cap detection and quantitation (NAD-capQ) approach that detects NAD caps released from the 5′-end of RNAs *en masse* (Supplementary Fig. 3a)^21^. Consistent with a deNADding function for Xrn1 in cells, a statistically significant 1.5-fold higher level of total NAD cap was detected in the *xrn1∆* strain relative to the WT strain (Fig. 3c).

### Xrn1 deNADding activity influences NAD-capped mitochondrial transcripts

Previous transcriptome-wide analyses of NAD-capped RNA in budding yeast identified distinct NAD-capped nuclear and mitochondrial transcripts^4,22^ with levels as high as 50% of select mitochondrial RNAs containing an NAD cap^22^. To determine whether the accumulation of endogenous NAD-capped RNAs was altered in the absence of Xrn1, NAD-capped RNAs were isolated by NAD capture^23^ and subjected to RT-qPCR analyses. We focused on a subset of previously identified NAD-capped nuclear- and mitochondrial-encoded mRNAs^4^. Importantly, although loss of *xrn1* did not change the steady state accumulation of the nuclear encoded NAD-capped RNAs tested, all the mitochondrially encoded NAD-capped transcripts tested were significantly elevated (Fig. 3d). Furthermore, we validated the qPCR results by an independent approach using boronate affinity electrophoresis for two transcripts, Cox2 and 21S rRNA^22^. This method allows visualization of distinct capped and uncapped RNA populations by specifically retarding the mobility of NAD-capped RNAs in the gel due to transient formation of diesters between immobilized boronic acid and the ribose moiety^22,24^. DNAzyme-mediated RNA cleavage was used to generate 5′-end-containing subfragments of defined length (Supplementary Fig. 3b). Northern blot analysis with the ^32^P labeled transcript-specific probes corroborated the NAD capture analyses for Cox2 and 21S rRNA with elevated NAD-capped RNA in the *xrn1∆* strain (Supplementary Fig. 3c). This observation prompted us to test whether Xrn1 is associated with mitochondria. In addition to its well characterized cytoplasmic localization^25^, Xrn1 has also been reported on cell membrane compartments called eisosomes that mark the sites of endocytosis during post-diauxic shift^26^. Gradient centrifugation purified mitochondria derived from a strain harboring Xrn1 with a C-terminus Strep-tag II was analyzed by Western blot analysis with an anti-Strep-tag antibody. As shown in Fig. 3e Xrn1 was detected with mitochondrial preparations suggesting Xrn1 can be mitochondrially associated.

Immunofluorescent colocalization was next used to more directly determine the localization of Xrn1. A knock-in strain of the green fluorescence protein (GFP) gene inserted in frame into the endogenous *XRN1* gene analogous to that reported in^27^ was generated within a strain background harboring red m-cherry fluorescent peptide fused to Edc3, a P-body marker^28^. Consistent with a previous report^27^, GFP tagged Xrn1 was functional and did not exhibit any growth phenotype when grown on YPG media (Supplementary Fig. 3d). Mitochondria were stained using Mitotracker® Deep Red FM dye. Visualization of Xrn1-GFP in conjunction with Edc3-mcherry and Mitotracker® Deep Red FM by confocal microscopy revealed four populations of Xrn1 foci (Fig. 3f). In the first, Xrn1 colocalized with the Edc3 P body marker, while in a second, Xrn1 foci were devoid of Edc3 indicating Xrn1 can be in distinct foci. A third population of foci contained an association of all three signals consistent with a previous report demonstrating the association of P-body foci with mitochondria in human cells^29^, while the fourth contained Xrn1 and mitochondrial network, but no Edc3-containing foci. Interestingly, we did not detect association of Edc3-containing P-body foci with mitochondria in the absence of Xrn1. These findings demonstrate the dynamic nature of Xrn1 localization within distinct foci.

**Fig. 4:**
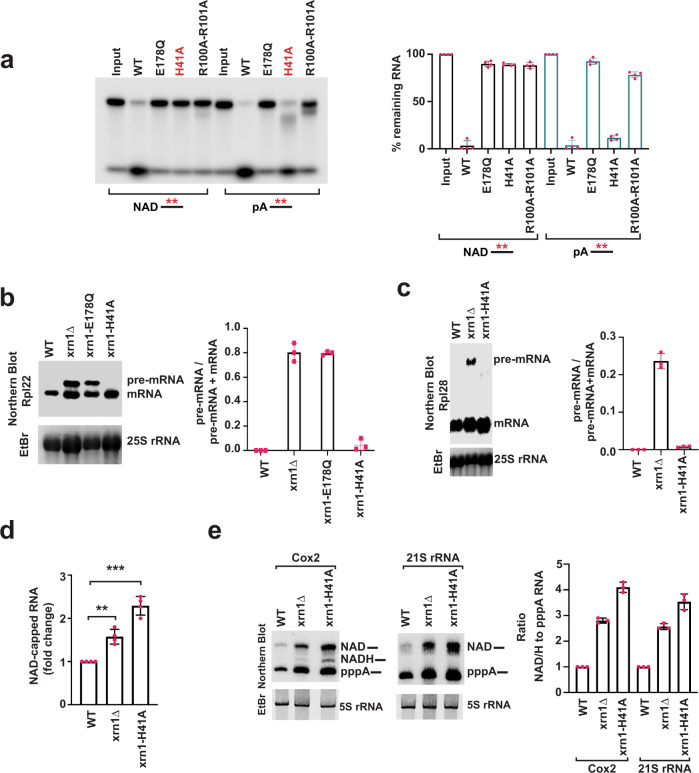
A conserved histidine residue in the catalytic pocket of Xrn1 is indispensable for NAD-capped RNA hydrolysis. Xrn1 mutational screen revealed that H41 is indispensable for NAD-capped RNA hydrolysis. **a** 30 nM recombinant WT or mutated Xrn1 from *K. lactis* was incubated with uniformly ^32^P-labeled 5′-monophosphate or NAD-capped RNA. The products were resolved by 15% 7 M urea PAGE gel. Quantitation from four independent experiments are presented on the right with the percent of full length RNA remaining. Error bars represent ±SD. **b** Northern Blot for Rpl22 mRNA in WT, *xrn1Δ, xrn1*-E178Q, and *xrn1*-H41A knock-in mutant is shown. Quantitation from three independent experiments are presented on the right with the mean ratio of Rpl22 pre-mRNA over total (pre-mRNA + mRNA). Error bars represent ±SD. **c** Northern Blot for Rpl28 mRNA in WT, *xrn1Δ* and xrn1-H41A knock-in mutant is shown. Quantitation from three independent experiments are presented on the right with the mean ratio of RPL28 pre-mRNA over total (pre-mRNA + mRNA) for the WT, *xrn1Δ* and *xrn1*-H41A. Error bars represent ±SD. **d** Total RNAs from WT, *xrn1Δ*, *xrn1*-H41A strains were subjected to the NAD-capQ assay to detect total level of cellular NAD-capped RNAs. Average from *n* = 4 independent experiments are shown with error bars denoting ±SEM. Statistical significance level was calculated by one-way ANOVA (*F* = 18.30, df = 2.209, *p* < 0.0001) with a Dunnett’s multiple comparison post hoc test, **p = 0.0036, ***p = 0.00013. **e** Northern Blot analysis of DNAzyme-generated 5′-end-containing subfragments of Cox2 and 21S rRNA from WT, *xrn1Δ* and *xrn1-*H41A grown in YPD (fermentation) media. Quantitation from three independent experiments with error bars representing ±SD are shown on the right. Source data are provided in Source Data File.

Using standard confocal microscopy with a resolution of ~200 nm shown in the left top panels of the Fig. 4g, a diffuse cytoplasmic distribution of Xrn1 with distinct foci was observed analogous to previous reports^30^. However, this level of resolution could not distinguish the extent of Xrn1 and mitochondrial colocalization. To further substantiate the Xrn1 and mitochondrial association, we performed super-resolution stochastic optical reconstruction microscopy (STORM) to visualize their localization at 10–20 nm resolution^31^. As shown with both a cross-section (left bottom panel) and Z-stacks (right panel) of Xrn1 and mitochondrial localization in Fig. 3g, the two signals are superimposable, indicating Xrn1 can be localized within mitochondria in yeast cells. Collectively, both the in vitro reconstitution and the in vivo analysis demonstrated a functional role for Xrn1 in regulating the fate of NAD-capped RNA in yeast mitochondria.

### A conserved histidine residue in the catalytic pocket of Xrn1 is indispensable for NAD-capped RNA hydrolysis

Structural and functional analyses of Xrn1 from *K. lactis* and *Drosophila melanogaster* have elucidated the molecular basis of the 5′P-specificity and the mechanism of processive RNA degradation^19,32^. The 5′P is embedded in a highly basic pocket lined with the side chains of the conserved amino acids K93, E97, R100, and R101 with His41 providing directionality to the decay process^32^ (Supplementary Fig. 4a). To determine whether the 5′P hydrolysis activity of Xrn1 can be uncoupled from the deNADding activity, a mutational screen was initiated. Structure guided point mutations were generated in the 5′P binding pocket and the catalytic residues assessed for their deNADding activity. Importantly, one residue, His41, was indispensable for deNADding while it still supported 5′P RNA decay activity (Fig. 4a, Supplementary Fig. 4b). To gauge the impact of H41A on deNADding in cells, a *xrn1-H41A* knock-in mutant strain was generated. Consistent with the in vitro analysis, the H41A mutant retained 5′P mediated exonuclease activity in cells as shown by its capacity to process its natural nuclear encoded substrate, Rpl22 precursor RNA^33^ (Fig. 4b). In contrast, the Rpl22 precursor RNA was detected at levels comparable to *xrn1Δ* when the catalytically inactive *xrn1*-E178Q knock-in mutant was used. Similar results were also obtained with a second Xrn1 target RNA (Rpl28 precursor mRNA)^27,34^, which was also efficiently degraded by the H41A mutant of Xrn1 (Fig. 4c). We next tested the consequence of a deNADding deficient Xrn1 on NAD-capped RNA levels in cells and detected an elevation of total NAD-capped RNA in the *xrn1Δ* strain and even greater elevation in the *xrn1-H41A* knock-in strain (Fig. 4d). The latter is consistent with the H41A protein still maintaining its 5’ monophosphate exonuclease activity and consequently leading to a higher relative level of NAD-capped RNA. Moreover, direct detection of the Cox2 and 21S rRNA mitochondrial transcripts by DNAzyme analysis revealed a four- and three-fold elevation of NAD-capped RNA in the *xrn1-H41A* strain, respectively (Fig. 4e). We conclude that H41 is a critical residue for the Xrn1-directed deNADding activity in cells and provides an avenue to directly determine the functional significance of Xrn1 deNADding in cells.

### Xrn1 maintains NAD-capped RNA in mitochondria

**Fig. 5:**
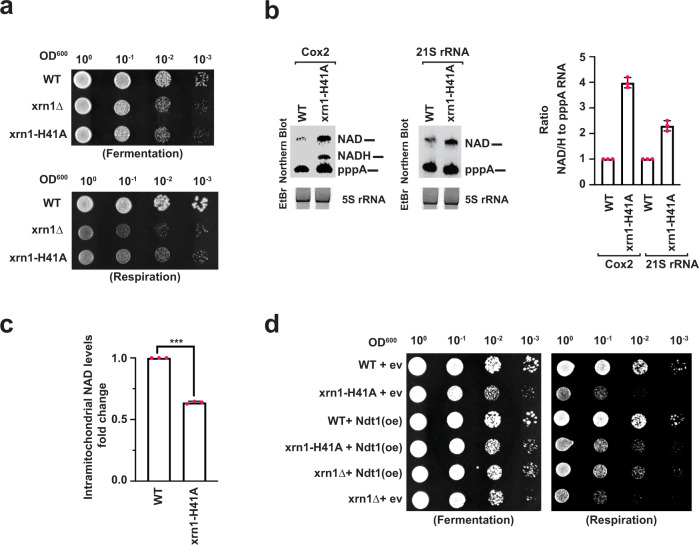
Xrn1 deNADding activity is critical in maintaining intramitochondrial NAD steady state levels. **a** Tenfold serial dilution of WT, *xrn1Δ* and *xrn1*-H41A were spotted onto solid YPD (Fermentation) and YPG (Respiration) plates and incubated at 30 °C. **b** Northern Blot analysis of DNAzyme-generated 5′-end-containing subfragments of Cox2 and 21S rRNA from WT, *xrn1Δ* and *xrn1*-H41A grown in YPG media with the quantitation on the right (Average of three independent experiments with error bars represent ±SD). Source data are provided in Source Data File. **c** Intramitochondrial NAD/NADH levels in WT and *xrn1*-H41A cells grown on YPG. Data are presented relative to the WT cells set to 1 and derived from three biological replicates (Average of three independent experiments with error bars represent ±SEM; two sided unpaired *t* test; ***p = 0.000001). Source data are provided in Source Data File. **d** Tenfold serial dilution of WT, *xrn1Δ* and *xrn1*-H41A harboring either an empty vector (ev)—without Ndt1 or a multicopy plasmid containing Ndt1 under a strong promoter—over expressed (oe) were spotted onto solid YPD and YPG plates and incubated at 30 °C.

The 5′-3′ exonuclease activity of Xrn1 is known to be necessary for growth on nonfermentable carbon sources in yeast resulting in a slow-growth phenotype in its absence^35^. We next determined whether Xrn1 deNADding activity contributed to the inhibitory growth on non-fermenting sugar utilizing the deNADding-deficient *xrn1-H41A* strain. As expected, all strains exhibited commensurate growth under fermentation growth parameters with glucose which would not require mitochondrial activity for growth (Fig. 5a). Interestingly, the deNADding compromised *xrn1-H41A* strain demonstrated a slow-growth phenotype comparable to the *xrn1* deletion strain on glycerol as the non-fermenting carbon source (Fig. 5a) where mitochondrial activity is required for cellular energetics. Importantly, a corresponding increase in NAD-capped RNA was also evident in *xrn1-H41A* mutant mitochondrial RNA (Fig. 5b), indicating a correlation between NAD-capped RNA and cellular growth when mitochondrial function was essential.

To determine whether levels of NAD-capped RNA influenced overall mitochondrial NAD levels, NAD/H levels were determined from WT and *xrn1-H41A* mitochondria derived under respiring growth conditions. Our findings revealed a ~35% reduction of intramitochondrial NAD levels in the *xrn1-H41A* mutant strain (Fig. 5c). With NAD-capped RNAs constituting a significant fraction of mitochondrial RNA in yeast^22^, these data imply NAD-capped RNAs may function as a source of mitochondrial NAD. To further determine whether the observed slow growth phenotype of the *xrn1-H41A* strain was a consequence of reduced mitochondrial NAD levels, we overexpressed the main mitochondrial NAD transporter protein Ndt1, which leads to elevated intramitochondrial NAD^36^. As shown in Fig. 5d, overexpression of Ndt1 reduced the slow growth phenotype observed in both the *xrn1Δ* and *xrn1-H41A* strains demonstrating the lower levels of mitochondrial NAD are a contributing factor to the observed slow growth phenotype. We conclude Xrn1 can regulate mitochondrial NAD-capped RNA through its deNADding activity and may link RNA metabolism to more general mitochondrial health and potential mitochondriopathies.

## Discussion

Pyridine dinucleotides, particularly nicotinamide adenine dinucleotide (NAD) and its reduced form NADH are essential players in a variety of cellular processes, including energy metabolism, cellular signaling, and transcription^1^. Recent mass spectrometry^23,37,38^ and chemoenzymatic analysis^23^ of cellular transcripts in organisms representing all three kingdoms of life have revealed that these dinucleotides also serve as non-canonical 5′-end RNA caps^12^. Nevertheless, the precise role of these non-canonical caps remained unknown.

In the present study, we characterized cellular proteins from the yeast *S.cerevisiae* interacting with a 5′NAD cap using NAD cap RNA affinity purification (NcRAP) and mass spectrometry. Our data revealed that unlike the canonical m^7^G caps, the NAD cap do not interact with translational factors or proteins involved in cellular translation, suggesting that NAD-capped RNAs are unlikely to be translated in budding yeast, consistent with the lack of yeast extract supporting NAD-capped RNA translation in vitro^39^. Surprisingly, herein we revealed that highly conserved 5′-monophosphate (5′P)-specific 5′-3′ exoribonucleases, Xrn1 and Rat1 along with its interacting partner Rai1 (yeast homolog of human DXO) can associate with and hydrolyze 5′NAD-RNA in vitro. Notably, similar to the DXO/Rai1 family of proteins, both Xrn1 and Rat1 release intact NAD from the NAD-capped RNA and have the capacity to regenerate NAD.

Another unexpected outcome of our study was the influence of Xrn1 on NAD-capped mitochondrial RNAs. Although Xrn1 has been reported to colocalize with P-body markers^30^, there were no previous indication of mitochondrial association. Our high resolution microscopy demonstrates in addition to the association with P-body markers, Xrn1 can also colocalized with a mitochondrial marker in yeast suggesting the modulation of mitochondrial NAD-capped RNA by Xrn1 is direct rather than indirect. Moreover, standard confocal imaging also revealed association of P-bodies with mitochondria, but interestingly, only if Xrn1 was also associated. Association of P-bodies on the surface of mitochondria has previously been reported^29^, whether such an interaction is directed through Xrn1 remains to be determined. Future high-resolution analysis of all three structures simultaneous will be necessary to more precisely determine their relative localization.

Although our initial objective was to identify NAD cap-binding proteins, the prominent proteins associated with the NAD cap were RNA nucleases and not bona fide NAD cap-binding proteins. This finding is consistent with our previous report that the NAD cap functions as a tag to promote rapid RNA decay^3^. It is possible that eukaryotic cells are devoid of NAD cap-binding proteins and NAD-capped RNAs instead are recognized by RNA nucleases to rapidly clear the RNA. More refined attempts with extract derived from a stain lacking the three dominant NAD cap associated proteins, Xrn1, Rai1 and a conditional depletion of Rat1, may be required to detect additional bound proteins. In addition to Xrn1, we also demonstrated that Rat1 is capable of binding and hydrolyzing NAD-capped RNAs. Since Rat1 and Rai1 are primarily nuclear proteins^25^ it is likely that their activity is predominantly on nuclear NAD-capped RNAs distinct from Xrn1. A more holistic and comparative analysis of the NAD-capped RNAs in the mutant strains of these three deNADding enzymes will aid in understanding their distinct or overlapping NAD-capped RNA substrates.

Mutational analysis of the catalytic center of Xrn1 led to the identification of a conserved histidine residue—His41, which is indispensable for deNADding, but does not significantly affect 5′P hydrolysis. Consistent with the proposed contribution of H41 to enzyme processivity^32^, low levels of intermediates were detected with the H41A mutant protein. Nevertheless, the H41A protein was still capable of degrading RNA both in vitro^32^ (Fig. 4a) and in cells (Fig. 4b, c). Importantly, this residue allowed for the uncoupling of the deNADding activity of Xrn1 from the 5′P exoribonuclease activity and enabled a delineation of the physiological role of NAD-capped RNA in yeast. Moreover, based on the observations of the *xrn1-H41A* knock-in mutant cells, we suggest that the previously characterized slow growth phenotype of *xrn1∆* on non-fermenting sugar^35^ is likely due to the loss of deNADding activity and not its monophosphate directed RNA decay.

Identification of a mutation in H41 being able to support 5′P exoribonuclease activity was surprising, since two charged substitution within this residue were identified in a synthetic lethal screen with the 3′ exonuclease essential *ski2*Δ mutant^40^ suggesting these substitutions disrupt Xrn1 exonuclease activity. However, analysis of recombinant Xrn1 harboring either the H41D or H41R mutation reveals that only H41D disrupts 5′P exoribonuclease activity while H41R retains robust exonuclease activity (Supplementary Fig. 4b) suggesting a more complex contribution of H41 to Xrn1 function. Future structural analysis of the H41A apo protein and in complex with NAD cap will illuminate the mechanism by which it can support exonuclease activity but not deNADding.

The intracellular concentration of pyridine dinucleotides is tightly regulated in cells^41^. Interestingly, we observe ~35% reduction in the intramitochondrial NAD levels in *xrn1-H41A* strains grown in YPG (non-fermenting sugar). Since mitochondria are indispensable for respiration, a decrease in NAD levels in mitochondria result in a significant slow growth phenotype upon growth on YPG media. It is important to note that mitochondria of budding yeast lack enzymes necessary for NAD synthesis and primarily rely on NAD imported from the cytoplasm^42^. In addition, the availability of free NAD dictates levels of mitochondrial NAD-capped RNA synthesis^22^. However, the full extent of Xrn1-H41A on mitochondrial NAD levels remains unclear since NAD-capped RNAs likely constitute <1% of total mitochondrial NAD levels. Whether this indicates cytoplasmic H41A deNADding may also contribute to mitochondrial NAD levels or the pools of “free” NAD are a smaller fraction of the total NAD levels remains to be determined. Collectively, our data support a role of Xrn1 in the turnover of mitochondrial NAD-capped RNA that may contribute to mitochondrial NAD and cellular growth during respiration in budding yeast. A recent report in *Arabidopsis thaliana* reinforces the equilibrium between NAD caps and free NAD where stabilization of NAD caps leads to a decrease in free NAD and a compensatory increase in NAD production following stress^13^. Whether NAD molecules stored in the 5′ end of RNA are used in adverse scenarios like nutrient starvation or quiescence in mammalian cells and the contribution of Xrn1 in NAD levels will be an important area of future exploration.

## Methods

### Yeast growth and media

All *Saccharomyces cerevisiae* strains used in the present study were derived in the BY4741 strain background and their genotypes are listed in Supplementary Table 2. Yeast cells were grown at 30 °C either in YPD (Dextrose) or YPG (Glycerol) (1% w/v yeast extract, 2% w/v peptone, 2% w/v glucose/glycerol). For tenfold serial dilution growth assays, yeast cells were grown overnight in YPD or YPG medium and diluted first to an OD600 of 1 and then serially 1 to 10. From each diluted cultures, 5 µL was spotted onto a plate and incubated at 30 °C.

### Plasmids construction and PCR mediated site-directed Mutagenesis

Polymerase chain reaction (PCR) amplification was conducted from the genomic DNA of *K. lactis* using *XRN1* gene-specific primers listed in Table S2. The PCR product was gel purified and ligated into a pET-28 vector using In-Fusion® (Takara) cloning.

PCR mediated site-directed mutagenesis was used for introducing specific point mutations in the Xrn1 protein. PCR with *Single-Primer Reactions IN Parallel* (SPRINP)^43^ and high-fidelity-DNA polymerase was used. All the primers used for generating point mutations are listed in Supplementary Table 1 and all plasmids used are listed in Supplementary Table 2.

### In vivo site-directed mutagenesis using *Delitto Perfertto*

Mutation substitution in the *XRN1* gene was generated using *Delitto Perfetto*^44^. Briefly, a CORE (**CO**unterselectable marker and Reporter gene)—*URA3-kanMx* was introduced into the Xrn1 locus proximal to His41 (for H41A) and Glu178 (for E178Q) using 40‐nts flanking sequences for homologous recombination. In the second step, a PCR‐amplified fragment containing the mutation of interest was transformed into the strain to enable integration and excise CORE cassette in the process. All point mutations were validated by DNA sequencing.

For the generation of Xrn1-GFP strain, CORE was introduced between residues 235 and 236 using 40‐nts flanking sequences for homologous recombination, whereas for Edc3-mcherry the CORE was introduced adjacent to the stop codon. In the second step, a PCR‐amplified fragment using pVT100U-mtGFP (Addgene) for Xrn1 and pFA6a-link-yomCherry-CaURA3 (Addgene) for Edc3-mcherry as a template containing the GFP and mCherry, respectively was transformed into the strain to enable integration and excise CORE cassette in the process. All knock-ins were validated by DNA sequencing.

### Protein expression and purification

Recombinant proteins were expressed and purified using Nickel-NTA affinity purification. In brief, the ligated plasmid was transformed into chemical competent *E. coli* BL21 (DE3) cells by heat shock. 5 mL LB cultures containing 50 μg/mL kanamycin were inoculated with single colonies and grown overnight. These cultures were used to inoculate 1 L of the same medium and allowed to grow at 37 °C until an OD600 of 0.5. Protein expression was induced with 0.25 mM isopropyl D-thiogalactopyranoside (IPTG) and cells grown at 18 °C on a shaker for ∼18 h. Cells were collected by centrifugation, 5000 *g* for 15 min.

For protein purification, cell pellets were thawed and resuspended in ∼60 mL of buffer [25 mM HEPES (pH 7.5), 250 mM KCl, 10% glycerol, and 100 μM MnCl_2_] containing 0.4 mg/mL protease inhibitor phenylmethanesulfonylfluoride fluoride (PMSF). Cells were lysed by sonication, and the insoluble cell debris was separated by centrifugation. The Nickel-NTA affinity purification was performed as described previously^45^.

### In vitro transcription of NAD-capped RNAs

RNAs containing NAD and m^7^G cap structures were synthesized by in vitro transcription from a synthetic double stranded DNA template ɸ2.5-NAD-40 containing the T7 ɸ2.5 promoter and a single adenosine within the transcript contained at the transcription start site (Supplementary Table 1)^10^. For m^7^G-capped RNA, m^7^GpppA RNA Cap Structure Analog (New England Biolabs) was included in the transcription reaction. In vitro transcription was carried out at 37 °C overnight, using HiScribe^TM^ T7 High yield RNA Synthesis kit (New England Biolabs).

To generate ^32^P-labeled NAD-capped RNA, transcription was carried out in the presence of ^32^P-NAD (PerkinElmer) instead of ATP using ɸ2.5-AG-30 (Supplementary Table 1)^10^. To generate ^32^P uniformly labeled RNA, the transcription reactions were performed in the presence of [α-^32^P] GTP.

### NAD cap RNA affinity purification (NcRAP)

400 pmol of NAD and m^7^G capped RNAs were immobilized onto Dynabeads™ MyOne™ Streptavidin T1 (Invitrogen^TM)^) following manufacturer’s instructions. For NcRAP, yeast cells were grown in YPD at 30 °C. 2 L of exponentially grown yeast cultures were harvested and lysed using mortar and pestle in liquid nitrogen. The cell powder obtained after grinding was resuspended in lysis buffer (25 mM Tris-HCl pH 7.5, 150 mM CH_3_CO_2_K, 2.5 mM Mg (C_2_H_3_O_2_)_2_, 50 µM EDTA, 50 µM EGTA, 2.5% glycerol and 0.25% IPEGAL) along with complete EDTA-free Protease Inhibitor Cocktail (Roche) and incubated at 4 °C for 30 min. Samples were subsequently centrifuged for 10 min at 5000 *g* to remove the cell debris. The clarified protein lysate was then transferred to a fresh 15 mL Falcon tube and the protein concertation was determined using a Bradford assay. 5 mg of protein lysate was added to the pre-equilibrated (in 1X lysis buffer) NAD- or m^7^G-capped RNA coupled Dynabeads in 2 mL microcentrifuge tubes. These tubes were next incubated at 4 °C on an end to end rocker for 90 min. The beads were washed for at least 5 times with 1X lysis buffer, and the bound proteins were eluted with 2X Laemmli buffer (Bio-Rad Laboratories). The samples were incubated at 85 °C for 5 min and were next run on Novex™ WedgeWell™ 4–20%, Tris-Glycine, Protein Gel (Thermo Fisher Scientific). The protein gel was stained with SYPRO Ruby (Bio-Rad Laboratories) as per manufacturer’s instructions and specific bands were sliced and sent for mass spectrometry based identification. In parallel we also analyzed the entire eluate using mass spectrometry. A complete list of identified proteins are provided in Supplementary Data 1.

### Liquid chromatography-tandem mass spectrometry (LC-MS/MS) for protein identification

Mass spectrometry was carried out at Biological Mass Spectrometry Facility of Robert Wood Johnson Medical School and Rutgers, The State University of New Jersey. Each gel band was subjected to reduction with 10 mM DTT for 30 min at 60 C, alkylation with 20 mM iodoacetamide for 45 min at room temperature in the dark and digestion with 0.2 µg trypsin (sequencing grade, Thermo Scientific Cat#90058), and incubated overnight at 37 °C. Peptides were extracted twice with 5% formic acid, 60% acetonitrile and dried under vacuum.

Samples were analyzed by in-gel digestion and by LC-MS using Nano LC-MS/MS (Dionex Ultimate 3000 RLSCnano System, Thermo Fisher) interfaced with Eclipse (Thermo Fisher) and loaded onto a fused silica trap column (Acclaim PepMap 100, 75 μm × 2 cm, Thermo Fisher). After washing for 5 min at 5 µl/min with 0.1% TFA, the trap column was brought in-line with an analytical column (Nanoease MZ peptide BEH C18, 130A, 1.7  μm, 75  μm × 250 mm, Waters) for LC-MS/MS. Peptides were fractionated at 300 nL/min using a segmented linear gradient 4–15% B in 30 min (where A: 0.2% formic acid, and B: 0.16% formic acid, 80% acetonitrile), 15–25%B in 40 min, 25–50%B in 44 min, and 50-90%B in 11 min. The next run starts following a return of Solution B to 4% for 5 min. Mass spectrometry data were acquired using a data-dependent acquisition procedure with a cyclic series of a full scan with a resolution of 120,000 followed by MS/MS (HCD, relative collision energy 27%) of the 20 most intense ions and a dynamic exclusion duration of 20 s.

The peak list of the LC-MSMS was generated by Thermo Proteome Discoverer (v. 2.1) into MASCOT Generic Format (MGF) and searched against the uniprot human fasta database and a database composed of common lab contaminants (CRAP) using in house version of X!Tandem (GPM Fury ^46^). Search parameters are as follows: fragment mass error, 20 ppm; parent mass error, ±7 ppm; fixed modification, carbamidomethylation on cysteine; variable modifications: methionine monooxidation for the primary search, asparagine deamination, tryptophan oxidation and dioxidation, Methionine dioxidation, and glutamine to pyro-glutamine were considered at the refinement stage. Protease specificity: trypsin C-terminal of R/K unless followed by P with 1 missed cleavage during the preliminary search and 5 missed cleavages during refinement. Minimum acceptable peptide and protein expectation scores were set at 10^−2^ and 10^−4^, respectively. The overall peptide false-positive rate^47^ was 0.07%. We applied 15 counts as a cutoff to distinguish nonspecific interactors and contaminants. These data are presented as a separate sheet (Cutoff-15) in Supplementary Data 1 along with the raw data.

### RNA in vitro deNADding assay

The ^32^P-NAD-cap labeled and [α-^32^P]GTP uniformly labeled NAD-capped RNAs were incubated with the indicated amounts of recombinant protein in NEB buffer 3 (100 mM NaCl, 50 mM Tris-HCl, 10 mM MgCl_2_, pH 7.9). Reactions were stopped by heating at 65 °C for 5 min. Decapping products were resolved by polyethyleneimine (PEI)-cellulose thin-layer chromatography (TLC) (Sigma-Aldrich) and developed in 0.45 M (NH_4_)_2_SO_4_.

For in vitro 5′-end RNA decay, cell extract corresponding to 2 µg of cellular protein from WT or *xrn1Δ* strains were incubated with [α-^32^P]GTP uniformly labeled NAD and m^7^G capped RNAs immobilized onto Dynabeads™ MyOne™ Streptavidin T1 (Invitrogen^TM)^) in NEB buffer 3 (100 mM NaCl, 50 mM Tris-HCl, 10 mM MgCl_2_, pH 7.9).

### NAD-cap detection and quantitation (NAD-capQ)

Yeast strains were grown in YPD media at 30 °C according to standard protocols. All strains were harvested for the experiments at exponential phase (OD600 ~1) and total RNA was isolated using the acidic hot phenol method^48^. To remove residual free NAD, purified RNAs were dissolved in 10 mM Tris-HCl (pH 7.5) containing 2 M urea. Samples were incubated 2 min at 65 °C and immediately precipitated with isopropanol in the presence of 2 M ammonium acetate. NAD-capQ was carried out as previously described^21^. Briefly, 50 μg of total RNA was digested with 2 U of Nuclease P1 (Sigma-Aldrich) in 20 μL of 10 mM Tris (pH 7.0), 20 μM ZnCl_2_ at 37 °C for 1 h to release 5′-end NAD. The control samples lacking Nuclease P1 were prepared by incubating 50 μg of RNA treated with the same reaction condition. The NADH standard curve was generated for each experiment in the same buffer condition as above for assays containing nuclease P1. Following digestion with nuclease P1, 30 μL of NAD/NADH Extraction Buffer (NAD/NADH Quantitation Kit, Sigma-Aldrich) was added to each sample. In the second step, 50 μL samples were used in a colorimetric assay according to the manufacturer’s protocol (NAD/NADH Quantitation Kit, Sigma-Aldrich) as described^21^.

### Isolation of NAD-capped RNAs by NAD capture and RNA quantitation

Total RNA from yeast strains grown in YPD media was isolated with the acidic hot phenol method^48^ and treated with DNase (Promega) according to the manufacturers’ protocols. NAD-capped RNAs were isolated using the NAD-RNA capture protocol^23^ with minor modifications. NAD-capture was carried out with 50 µg total RNA treated with 10 µL 4-pentyn-1-ol (Sigma-Aldrich) and 3 U Adenosine diphosphate-ribosylcyclase (ADPRC) in 100 µL reaction containing 50 mM HEPES, 5 mM MgCl_2_ (pH 7) and 40 U RNasin^®^ Ribonuclease Inhibitor (Promega) at 37^o^ C 60 min. The reaction was stopped with phenol/chloroform extraction and RNAs were precipitated with ethanol in the presence of 2 M ammonium acetate. RNAs with a 5′-end NAD were biotinylated by treatment with a copper-catalyzed azide-alkyne cycloaddition (CuAAC) reaction by incubating with 250 µM biotin-PEG3-azide, freshly mixed 1 mM CuSO4, 0.5 mM THPTA, 2 mM Sodium Ascorbate in 100 µL reaction with 50 mM HEPES, 5 mM MgCl_2_ (pH 7) and 40 U RNasin^®^ Ribonuclease Inhibitor (Promega) at 30 °C for 30 min. RNA was precipitated with ethanol in the presence of 2 mM EDTA and 2 M ammonium acetate. Biotinylated NAD-capped RNAs were captured by binding to 20 µL streptavidin magnet beads (Nvigen) at room temperature with gentle rocking for 1 h in 100 µL binding buffer (1 M NaCl, 10 mM HEPES (pH 7.5) and 5 mM EDTA). The beads were washed three times with buffer containing 8 mM Urea, 50 mM Tris-HCl (pH 7.4), 0.1% IPEGAL). To elute biotinylated NAD-capped RNAs, beads were resuspended in 100 µL of above buffer, heated to 95 °C for 5 min and RNA precipitated with ethanol in the presence of glycogen and 2 M ammonium acetate. Captured NAD-RNAs were dissolved in 20 µL H_2_O and reverse transcribed with M-MLV reverse transcriptase, random hexamers, and oligo (dT) (Promega) according to the manufacturer’s instructions. qRT-PCR was performed with the primers listed in Supplementary Table 1. qRT-PCR was carried out on QuantStudio 3 Real-Time PCR System (Thermo Fisher Scientific) with iTaq SYBR Green Supermix (Bio-Rad Laboratories).

### Boronate affinity electrophoresis and Northern Blotting analysis of in vivo NAD-capped transcripts after DNAzyme-mediated RNA cleavage

For analysis of NAD and NADH-capping during fermentation, yeast strains were grown in YPD and for respiration in YPG media at 30 °C. RNA was isolated from cells at the exponential phase (OD600 ~1) with the acidic hot phenol method^48^ and treated with DNase (Promega). 50 μg of total cellular RNA was incubated with 1 μM of the corresponding DNAzyme ((Supplementary Table 1) in 50 μL reaction containing 10 mM Tris pH = 8.0, 50 mM NaCl, 2 mM DTT and 10 mM MgCl_2_. Samples were heated at 85 °C for 2 min and slow cooled to 37 °C. MgCl_2_ was added to a final concentration of 10 mM and incubated for 60 min at 37 °C. Reactions were terminated with 100 μL of stop solution (50 mM Tris pH 8.0, 20 mM EDTA, and 0.1 µg/mL glycogen) and RNA was precipitated with 500 μL ethanol by incubating for 30 min at −80 °C followed by centrifugation for 30 min at 16,000 *g* at 4 °C. The supernatant was removed, and the pellet resuspended in H_2_O.

To analyze NAD-capping of DNAzyme-generated fragments of mitochondrial RNAs, 25 μg of cleaved RNA was separated by electrophoresis on 8% urea polyacrylamide gels supplemented with 0.3% 3-acrylamidophenylboronic acid (Boron Molecular). RNA was transferred to positively charged Nylon transfer membrane (GE Healthcare Life Sciences), immobilized by UV crosslinking, and incubated with a ^32^P-labeled oligodeoxyribonucleotide probe complementary to the 5′-end fragments of target RNAs (Supplementary Table 1). The probes were labeled using T4 polynucleotide kinase (New England BioLabs) and [γ-^32^P] ATP (Perkin Elmer). Reaction products were visualized with Amersham Typhoon RGB Biomolecular Imager (GE Healthcare Life Sciences) and bands corresponding to uncapped and NAD- or NADH-capped DNAzymes-generated fragments were quantified with IQ TL image analysis software.

### Isolation of mitochondria and quantification of intramitochondrial NAD levels

Mitochondria were isolated using density-gradient centrifugation as previously described^49^. Seven grams of exponentially grown cells were used to retrieve pure mitochondria from both YPD (fermentation) and YPG (respiration) cultures. Cells were first treated with Zymolyase (Sunrise Science Products) to generate spheroplasts, which were next lysed using a homogenizer. The unbroken cells, nuclei, and cell debris were next pelleted by three consecutive centrifuges—first for 5 min at 1500 *g* at 4 °C, second at 3000 *g* for 5 min and the third one at 12,000 *g* for 15 min. The resulting supernatant was next layered onto a sucrose gradient (15–60%) and purified mitochondria were isolated using density gradient centrifugation (134,000 *g* for 1 h). The intact mitochondria forms a brown band at the 60–32% sucrose interface. Mitochondrial preparation purity was assessed by Western blot analysis Anti-Cox2 (Anti-MTCO2 antibody [4B12A5] (ab110271)), and anti-Pgk1 ((PA5-28612) Invitrogen) antibodies were used to detect the respective proteins. For assessing the mitochondrial association of Xrn1, Xrn1-Strep-tag II strain was used. The Xrn1-Strep-tag II strain was generated using homologous recombination of a PCR product amplified from a plasmid pTF277 (Addgene) containing 40‐nts flanking sequences for homologous recombination. Xrn1 was detected using Strep Tag Monoclonal Antibody (GT517) (Invitrogen). A dilution of 1:2000 of all primary antibodies in PBST were used for Western Blotting. Secondary antibodies were diluted at 1:10,000.

Mitochondrial NAD levels were quantified from three independent mitochondrial preparations from both WT and *xrn1-H41A* strains using NAD/NADH Quantitation Kit (Sigma-Aldrich). Mitochondrial extract corresponding to 10 µg of protein from both WT and *xrn1-H41A* were used to determine NAD levels as per instructions provided in the kit.

### Confocal and STORM Imaging of yeast cells

Yeast cells were grown to the early exponential growth phase (OD600 nm = 0.4–0.7) in synthetic complete medium containing 2% glucose. Confocal microscopy was performed without fixing the cells. Mitotracker^®^ Deep Red FM (Cell Signaling Technology) was used for labeling mitochondria. Prior to imaging, 200 nM of Mitotracker^®^ Deep Red FM (Cell Signaling Technology) was added to the media and the cells were incubated for 30 min, after which cells were washed twice to remove excess of the dye. For super resolution STORM microscopy, mito-RFP^50^ was used for labeling mitochondria. Here cells were fixed with 3.7% formaldehyde in the growth medium at room temperature (RT, 20 min) and washed with 1X phosphate-buffered saline (PBS) with sorbitol (137 mM NaCl; 3 mM KCl; 8 mM Na2HPO4; 1.5 mM KH2PO4; 10% (w/v) sorbitol; pH 7). Cell walls were partially removed by zymolase treatment (15 min, 30 °C). Cover glass (#1.5 thickness) (Electron Microscopy Sciences) was treated with 1 M HCl extensively rinsed with distilled water and air dried. Cover glass was then treated with 100 µg/ml of poly-d-lysine for 2 h at RT, washed with 1X PBS and dried overnight. Cells were allowed to attach onto the treated cover glass before incubating with blocking solution (1X PBS/sorbitol, 5% bovine serum albumin (BSA) and 0.1% Triton X-100) for 1 h at RT. Rabbit anti-RFP (Rockland) and a mixture of mouse anti-GFP 8H11, 12E6 and G1 (Developmental Studies Hybridoma Bank) were incubated with the samples overnight at 4 °C. After removing the primary antibody solution, samples were washed with 1X PBS/sorbitol, 1% BSA, 0.05% Triton X-100. Secondary antibodies containing goat anti-mouse F (ab)_2_ Atto 488 and goat anti-rabbit Alexa Fluor 647 were incubated in blocking solution for 1 h at RT. Secondary antibody solution was removed and samples were washed with 1X PBS/sorbitol, 1% BSA, 0.05% Triton X-100. Samples were mounted on imaging buffer^51^ containing: 50 mM Tris·base, pH 8.0, 10 mM NaCl, 10% (wt/vol) glucose, 100 mM monoethanolamide (MEA), 40 μg/mL catalase, and 0.5 mg/mL of glucose oxidase. MEA was used as a thiol compound to facilitate photo switching of dyes. Catalase and glucose oxidase used an oxygen scavenger system to reduce photo bleaching. Primary antibodies, Rabbit anti-RFP (Rockland), mouse anti-GFP 8H11, 12E6 and G1 (Developmental Studies Hybridoma Bank) were all diluted at 1:1000. Secondary antibodies were diluted at 1:5000.

STORM was set up on an Olympus IX73 with an excitation light source containing 405-nm, 470-nm, and 590-nm excitation wavelengths. An Olympus 60 × 1.3 N.A. UPlanSApo objective was used to acquire 512 × 512-pixel images using an Andor Zyla 4.2 sCMOS camera at 25 frames/s. A spinning disk was included in the emission light path to block out of focus light and retain fluorescence from a single optical plane. An IR focus lock system (zero drift correction) was used to ensure minimal drift in the *z*-axis. Software was used to correct for XY drift in the images by using the translational setting of the StackReg plugin from ImageJ. Fluorescent 10-nm beads were used to determine the resolution of the system. As a measure of resolution, the full-width half mean of single beads were measured at ∼10 nm.

Excitation light was adjusted to 0.2–1 mW at 470 nm and 0.2–1 mW at 590 nm to acquire a sparse subset of fluorophores. Intensities of excitation light were measured at the back of the objective. Streaming acquisition of images was sequentially acquired with 10–15 ms exposure time. On average, 10,000-50,000 images were acquired for each field of view. Images from a single focal plane were acquired. Blinking events were identified by the change in fluorescence at individual pixel regions between frames. Images reconstruction, chromatic aberration correction and drift correction were done using ThunderSTORM^52^.

### Statistics and reproducibility

All statistical analysis and software used have been mentioned in the Figure Legends and Materials & Methods. GraphPad Prism version 8.2.0 was used for all statistical analysis. The SyproRuby stained gels for NcRAP experiments shown in Fig. 1b and Supplementary Fig. 1b are representative figures that were carried out from three independent affinity purification experiments. The panels shown for in vitro RNA decay experiments in Fig. 2b, c, and in Supplementary Figs., 2a–c, f, and 4b are representative of three independent experiments. TLC experiments in Fig. 2e and Supplementary Fig, 2e are representative of three independent experiments.

### Reporting summary

Further information on research design is available in the Nature Research Reporting Summary linked to this article.

## Supplementary information

Supplementary Information

Description of Additional Supplementary Files

Supplementary Data 1

Reporting Summary

## Data Availability

The data supporting the findings of this study are available from the corresponding authors upon reasonable request. Mass Spectrometry is deposited at MassIVE under Accession code MSV000087605. Source data for the figures and supplementary figures are provided as a Source Data file. Source data are provided with this paper.

## Source data

Source Data
