# Supplementary material for: Xrn1 is a deNADding enzyme modulating mitochondrial NAD-capped RNA
Source: Nat Commun. 2022 Feb 16;13:889. doi: 10.1038/s41467-022-28555-7 (PMC8850482; doi:10.1038/s41467-022-28555-7)
Supplement: Supplementary file 1 — Supplementary Information [file 41467_2022_28555_MOESM1_ESM.pdf]

Xrn1 is a deNADding Enzyme Modulating Mitochondrial NAD-capped RNAs

Sunny Sharma, Jun Yang, Ewa Grudzien-Nogalska, Jessica Shivas, Kelvin Y. Kwan and Megerditch Kiledjian*

Department of Cell Biology and Neuroscience, Rutgers University, Piscataway, NJ 08854, USA

*Correspondence should be addressed to:
Megerditch Kiledjian; kiledjian@biology.rutgers.edu

Supplementary Information

Supplementary Figures

a

		% of total bands in the lane			
		WT		xrn1Δ	
Gel Band	Protein	m^7G--	NAD--	NAD--	m^7G--
1)	eIF4G1	6.7%			5.3%
2)	eIF4G2	17.5%			16.5%
3)	Sto1	9.1%			14.3%
4)	eIF4E	7.6%			7%
5)	Cbc2	8.1%			7.3%
6)	Xrn1		25%	0%	
7)	Rat1		6.2%	15.3%	
8)	Rai1		2.1%	8%	

b

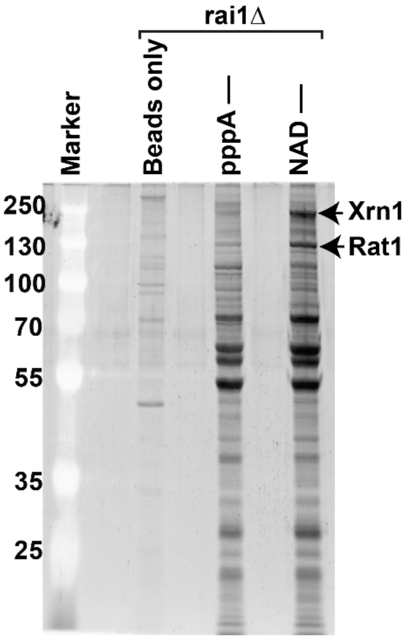

Supplementary Figure 1.

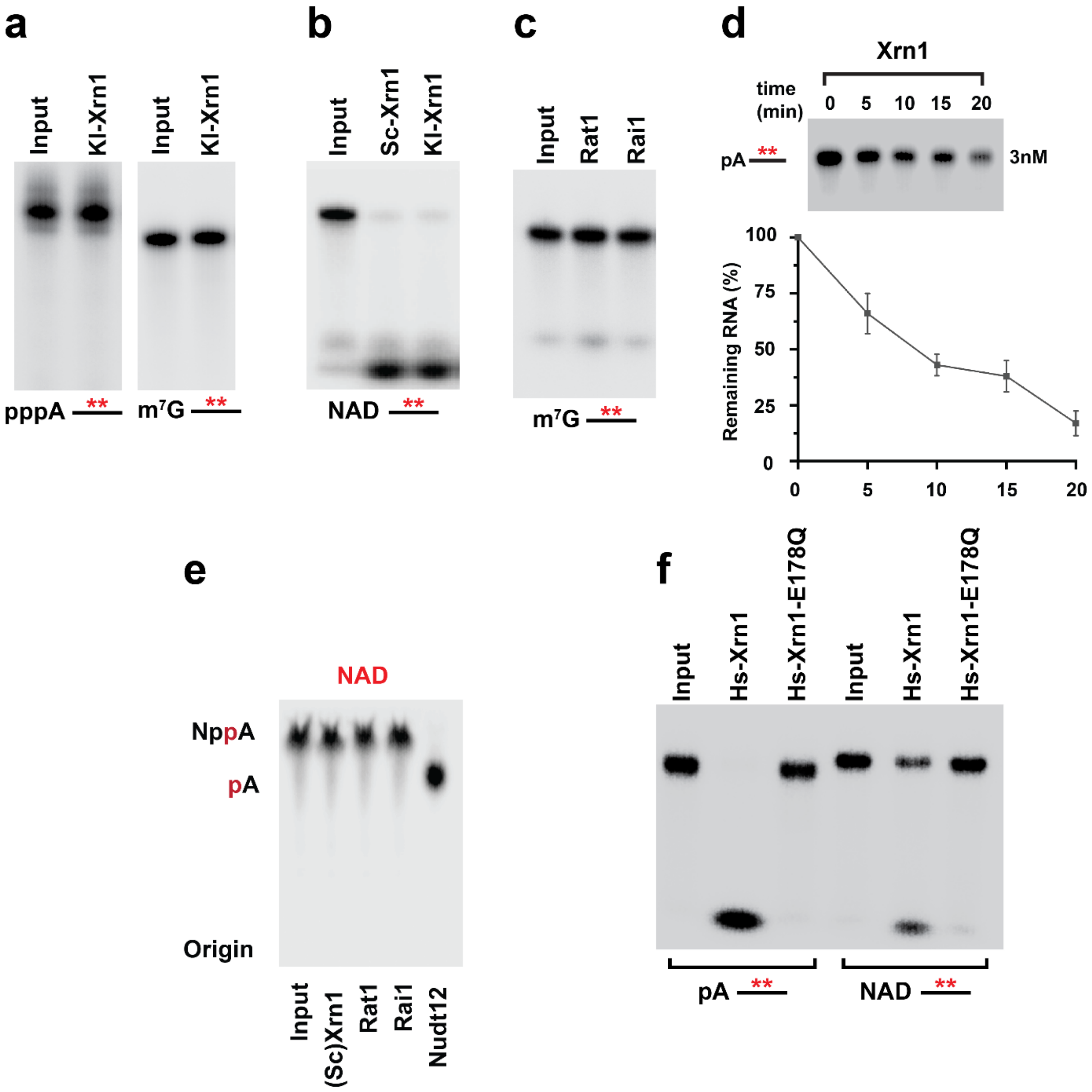

Supplementary Figure 2.

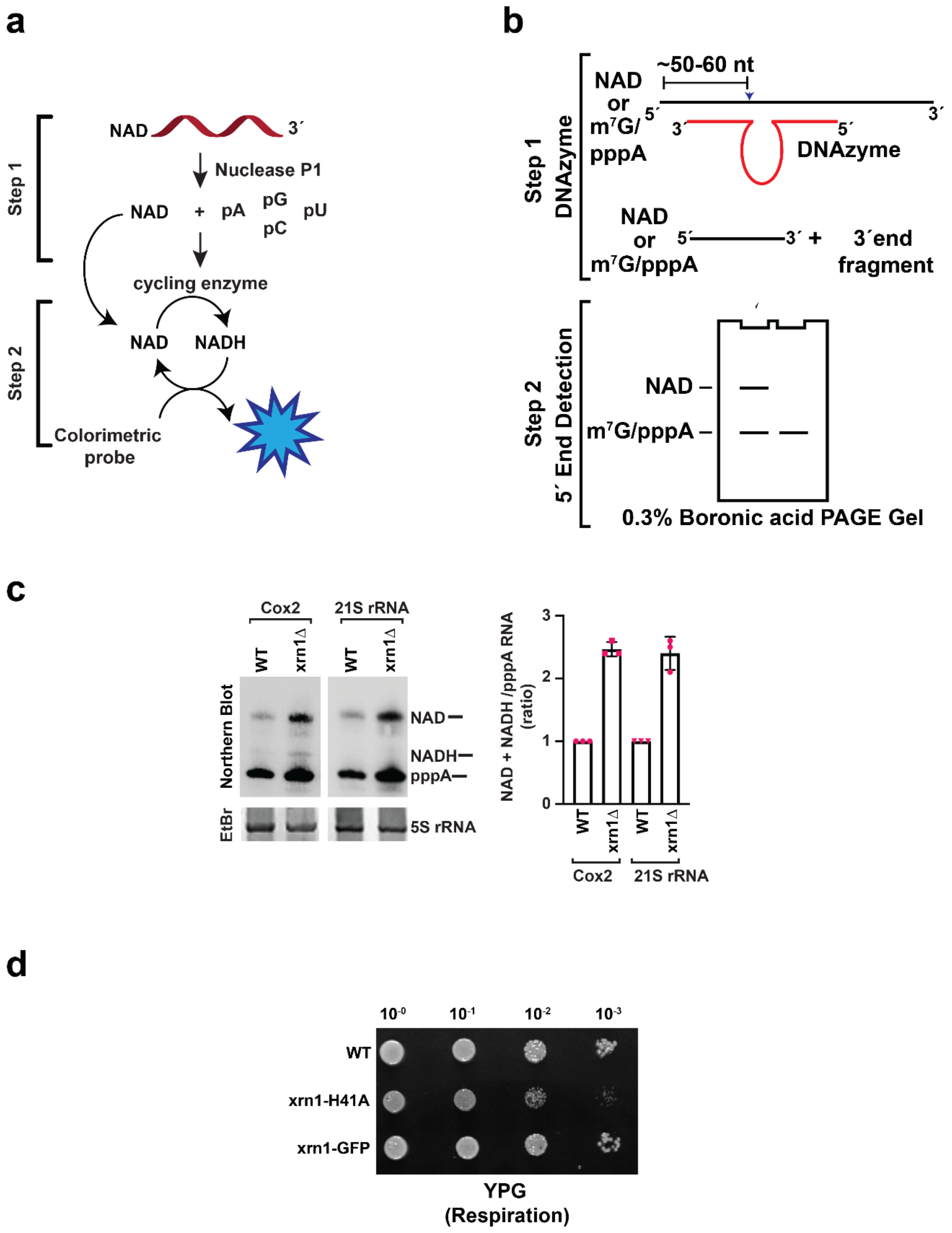

Supplementary Figure 3.

a

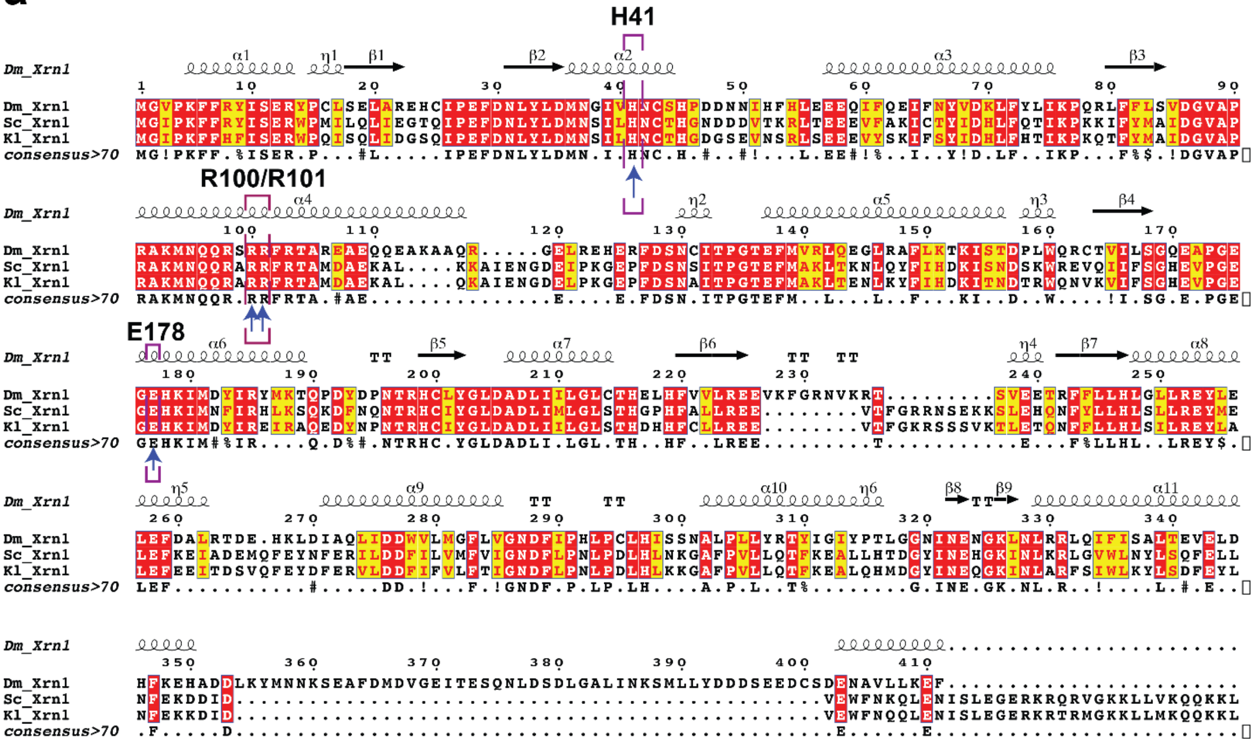

b

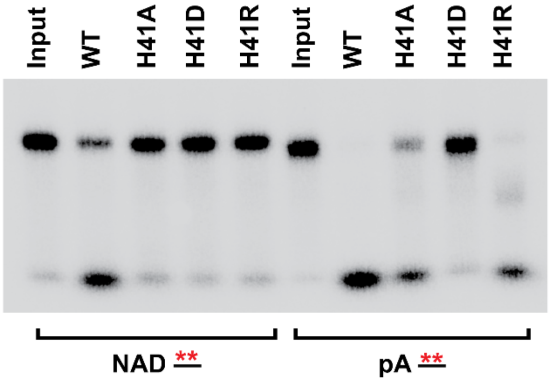

Supplementary Figure 4.

Supplementary Figure 1. (a) Quantification for the bands in panel b in each lane is presented as a percentage of the total band intensities. Here, only predominant bands were considered for the calculation and the lower two bands common to all the lanes are ignored. (b) Identification of proteins bound to the NAD cap in the absence of Rai1. Protein derived from a strain lacking Rai1 (rai1Δ) were captured by NcRAP and eluates detected by SYPRO Ruby following resolution on 10% SDS-PAGE gel. Affinity purification with 5′-end triphosphorylated RNA (pppA---) or NAD-capped RNA (NAD---) are shown.

Supplementary Figure 2. Xrn1 hydrolyzes NAD-capped RNA but not 5′-triphosphorylated or m7G-capped RNA. (a) Recombinant *K. lactis* Xrn1 (30 nM) was incubated with uniformly ^{32}P-labeled 5′-triphosphorylated (pppA) or m^{7}G-capped RNA. Products were resolved by 15% 7M urea PAGE. (b) Recombinant Xrn1 from *K. lactis* or *S. cerevisiae* (30 nM) were incubated with NAD-capped RNA uniformly ^{32}P-labeled and the products were resolved as in panel a. Source data are provided in the Source Data File. (c) *S. pombe* Rat1 (60 nM) or Rai1 (25 nM) were incubated with uniformly ^{32}P-labeled RNA containing a 5′-end m^{7}G cap and products resolved as in panel a. (d) Time-course decay analysis of uniformly ^{32}P-labeled monophosphate RNA (9pmol) with the indicated amount of Xrn1 (~45 fmol) protein are shown. Quantitation of RNA remaining is plotted from n=3 independent experiments with SD denoted by error bars (e) ^{32}P-labeled free NAD was incubated with *S. cerevisiae* Xrn1 (30nM), *S. pombe* Rat1 (60 nM), Rai1 (25 nM), or mouse Nudt12 (50 nM). The reaction products were resolved by polyethyleneimine (PEI)-cellulose thin layer chromatography developed in 0.45M $(NH_4)_2SO_4$. (f) Recombinant human Xrn1 (HsXrn1) (60 nM) was incubated for 45 minutes with uniformly ^{32}P-labeled NAD-capped RNA, and the products were resolved as in panel a.

Supplementary Figure 3. NAD-capQ and DNAzyme. (a) Schematic illustration of NAD-capQ as described in [21]. (b) Schematic representation of DNAzyme-mediated RNA cleavage coupled to Northern Blot detection of NAD-capped RNAs. (c) Northern Blot analysis of DNAzyme-generated 5′-end-containing Cox2 and 21S rRNA subfragments detected with ^{32}P-labeled transcript-specific probes are shown with the corresponding quantitation plotted on the right. Data are derived from n=3 independent experiments with error bars representing ±SD. Source data are provided in the Source Data File. (d) Tenfold serial dilution of WT, xrn1-H41A and xrn1-GFP were spotted onto solid YPG (Respiration) plates and incubated at 30°C.

Supplementary Figure 4. Xrn1 sequence alignment. (a) Amino acid sequences corresponding to the amino termini of Xrn1 from *D. melanogaster* (Dm), Homo sapiens (Hs), *S. cerevisiae* (Sc) and *K. lactis* (Kl) were aligned using ClustalW2 (EMBL-EBI) and the alignment file was analyzed with EsPript 3.2. Amino acid identity is marked in red and similarities are represented in yellow. The catalytically active residues are highlighted with black rectangles. (b) 60 nM recombinant WT or different point mutated Xrn1 for H41 residue-H41A, H41D, and H41R, from *K. lactis* was incubated with uniformly ^{32}P-labeled 5′-monophosphate or NAD-capped RNA. The products were resolved by a 15% 7M urea PAGE gel.

Supplementary Table 1. Oligonucleotides used in the present study

NAME	SEQUENCE 5'->3'	PURPOSE
ϕ2.5-NAD-40	CAGT**AATACGACTCACTATT**AGTTGGTGGTTGTTGTGTGTTTGTGGTTGGTTTGTTTGGC	In vitro transcription (T7 promoter sequence is highlighted)
ϕ2.5-AG-30	CAG**TAATACGACTCACTATT**AGCCCTCTCTTCCTTCCTTCCTCCTTTCCT	In vitro transcription (T7 promoter sequence is highlighted)
SP1-KLXRN1-H41A-F	gaattctattttgGCtaattgtacgcatggtgatg	For site directed mutagenesis (**pET-klxrn1-H41A)**
SP2-KLXRN1-H41A-R	catgcgtacaattaGCcaaaatagaattcatatcc	For site directed mutagenesis (**pET-klxrn1-H41A)**
SP7-KLXRN1-R100/101A-F	caacaaagagctGCtGCattcagaactgccatggatg	For site directed mutagenesis (**pET-klxrn1-R100/101A**
SP8-KLXRN1-R100/101A-R	catggcagttctgaatGCaGCagctctttgttggttcatc	For site directed mutagenesis (**pET-klxrn1-R100/101A)**
PET-HXRN1(1-1190)-A1	GGTGCCGCGCGGCAGCCATATGggagtccccaagttttacagatg	For generating pET-HsXrn1
PET-HXRN1(1-1190)-A2	CTCGAGTGCGGCCGCAAGCTcattttacgatggctgtcaacttctg	For generating pET-HsXrn1
PET-HXRN1-E178Q-A3	gaaggaCagcataaaatcatggaatttatc	For site directed mutagenesis (**pET-Hsxrn1- E178Q)**
PET-HXRN1-E178Q-A4	gattttatgctGtccttctccaggagtctc	For site directed mutagenesis (**pET-Hsxrn1- E178Q)**
P.I-XRN1-H41	CTTCAGTTAATCGCTTGGTTACATCATCGTCGTTACCATGCGTACAATTATGttcgtacgctgcaggtcgac	For Delitto Perfertto
P.II-XRN1-H41	CACAGATTCCTGAGTTTGATAACTTATACCTGGATATGAATTCGATTTTAtagggataacagggtaatCCGCGCGTTGGCCGATTCAT	For Delitto Perfertto
P.I-XRN1-E178	GGGATTTTAAATGCCTTATAAAGTTCATGATCTTGTGTTCttcgtacgctgcaggtcgac	For Delitto Perfertto
P.I-XRN1-E178	GTGCAAATCATATTTTCTGGCCATGAAGTTCCAGGTGAAGGTtagggataacagggtaatCCGCGCGTTGGCCGATTCAT	For Delitto Perfertto
PTF277-STREP-A1	GCAATGCTGCTGACCGTGATAATAAAAAAGACGAATCTACTgagaatttatacttccaag	For generating xrn1-Strep-tagII
PTF277-STREP-A2	AAAGTAACCTCGAATATACTTCGTTTTTAGTCGTATGTTCTAgcttatttagaagtggcg	For generating xrn1-Strep-tagII
P.DE-XRN1	ATATAACAGTTGCAGCTTGC	Diagnostic PCR
P.DI	ACGAGAACGGATGTAAGCATCACC	Diagnostic PCR
P.UE-XRN1	GATTCATCTTGGCACGAGGG	Diagnostic PCR
P.UI	GAGCAATGAACCCAATAACGAAATC	Diagnostic PCR

21S-NB-2	CTATATAATAAATATTTCAAATCTATTATTCT AC	Northern Blot probe for 21S rRNA
COX2-NB-2	ATCTTAACCTTTAGACTCTTTTGTCTATTTATA ATATGT	Northern Blot probe for Cox2
21S-DZ	ACTCCATGATTAGGCTAGCTACAACGACTCTT TAAATCT	DNAzyme for 21S
COX2-DZ	TCTTAATAAATCTAAGGCTAGCTACAACGAAT TTTAATAAATCTT	DNAzyme for Cox2
ATP6 F	TGC TTA AAG GAC AAA TTG GAG G	RT-PCR for ATP synthase subunit 6
ATP6 R	GAG CTG ATA ATG CAA ATG AGT ATG G	RT-PCR for ATP synthase subunit 6
ATP9 F	TGG AGC AGG TAT CTC AAC AAT T	RT-PCR for ATP synthase subunit 9
ATP9 R	TGA TGG GTT TCT TGA TAC ACC A	RT-PCR for ATP synthase subunit 9
EGD2 F	TAA CTA CGT TGT CTT CGG TGA A	RT-PCR for Enhancer of Gal4 DNA binding
EGD2 R	TAG ATG GCA TAA TAC CGC TAG C	RT-PCR for Enhancer of Gal4 DNA binding
COX1 F	GTG GTG GGA CAT TTT CAC TAT G	RT-PCR for Cytochrome c oxidase subunit 1
COX1 R	AAG CGA CAT AAT TTC ATC CTG C	RT-PCR for Cytochrome c oxidase subunit 1
COX2 F	TAG ATT CGT TGT AAC AGC TGC T	RT-PCR for Cytochrome c oxidase subunit 2
COX2 R	CTA CCA GGA GTA GCA TCA ACT T	RT-PCR for Cytochrome c oxidase subunit 2
LSM6 F	GGT AAG ACA GTG AAC GTC AAA C	RT-PCR for U6 snRNA-associated Sm-like protein
LSM6 R	TTA CTC TCG TAG TGT TCA GTG G	RT-PCR for U6 snRNA-associated Sm-like protein
RPA12 F	GCG CTA CTA TCA AGG AAA AGT G	RT-PCR for RNA polymerase I subunit RPA12
RPA12 R	CAA TTG TTG GTA CGG AAC TTG T	RT-PCR for RNA polymerase I subunit RPA12
21S F	ACCTTTTGTATAATGGGTCAGCA	RT-PCR for 21S rRNA
21S R	ACATCAACCTGTTCGATCGT	RT-PCR for 21S rRNA

Supplementary Table 2. Reagents used in the present study

REAGENT or RESOURCE	SOURCE	IDENTIFIER
**Yeast Strains**		
BY4741 (MATa; his3Δ1; leu2Δ0; met15Δ0; ura3Δ0)	Dharmacon	YSC1048
xrn1-H41A (BY4741; MATa; his3Δ1; leu2Δ0; met15Δ0; ura3Δ0; xrn1(C121G,A122C))	This study	NA
xrn1-E178Q (BY4741; MATa; his3Δ1; leu2Δ0; met15Δ0; ura3Δ0; xrn1(G532C))	This study	NA
Xrn1-GFP (BY4741; MATa; his3Δ1; leu2Δ0; met15Δ0; ura3Δ0; Xrn1-235-GFP))	This study	NA
Xrn1-GFP and Edc3-mCherry (BY4741; MATa; his3Δ1; leu2Δ0; met15Δ0; ura3Δ0; Xrn1-235-GFP;Edc3-mCherry))	This study	NA
Xrn1-Strep-tag II (BY4741; MATa; his3Δ1; leu2Δ0; met15Δ0; ura3Δ0; Xrn1-Strep-tagII))	This study	NA
**Plasmids**		
pET-KlXrn1 (A derivative pET26a(+) plasmid carrying KlXrn1(1,1245)-6xHis fusion protein)	Chang et al(2011)[9]	NA
pET-KlXrn1-E178Q (A derivative pET-KlXrn1 plasmid carrying mutant klxrn1-E178Q-6xHis fusion protein)	Chang et al(2011)[9]	NA
pET-klxrn1-H41A (A derivative pET-KlXrn1 plasmid carrying mutant klxrn1-H41A-6xHis fusion protein)	This study	NA
pET-klxrn1-H41D (A derivative pET-KlXrn1 plasmid carrying mutant klxrn1-H41D-6xHis fusion protein)	This study	NA
pET-klxrn1-H41R (A derivative pET-KlXrn1 plasmid carrying mutant klxrn1-H41R-6xHis fusion protein)	This study	NA
pET-klxrn1-K93A (A derivative pET-KlXrn1 plasmid carrying mutant klxrn1-K93A-6xHis fusion protein)	This study	NA
pET-klxrn1-Q97A (A derivative pET-KlXrn1 plasmid carrying mutant klxrn1-K97A-6xHis fusion protein)	This study	NA
pET-HsXrn1 (A derivative pET26a(+) plasmid carrying HsXrn1(1,1189)-6xHis fusion protein)	This study	NA
pET-HsXrn1-E178Q (A derivative pET26a(+) plasmid carrying mutant HsXrn1-E178Q(1,1189)-6xHis fusion protein)	This study	NA
pET-klxrn1-R100/101A (A derivative pET-KlXrn1 plasmid carrying mutant klxrn1-R100A,R101A-6xHis fusion protein)	This study	NA
pVT100U-mtGFP	Addgene	#45054
Mito-RFP (A derivative of pVT100U-GFP plasmid carrying RFP in place of GFP)	This study	NA
pFA6a-link-yomCherry-CaURA3	Addgene	#44876
pTF277	Addgene	#44094
**Chemicals, Peptides, and Recombinant Proteins**		
Xrn1	NEB	M0338S
Nuclease P_1 from *Penicillium citrinum*	Sigma-Aldrich	N8630-1VL
RQ1 RNase-Free DNase	Promega	M6101
Phenol/Chloroform/Isoamylalcohol pH4.3	Fisher Scientific	BP1754I400
T7 RNA polymerase	Promega	P2075
T4 DNA Ligase	Promega	M1801

Recombinant RNasin Ribonuclease Inhibitor	Promega	N2515
ADP-ribosylcyclase (ADPRC)	Sigma-Aldrich	A9106-1VL
4-pentyn-1-ol	Sigma-Aldrich	302481-5G
Azide-PEG3-biotin conjugate	Sigma-Aldrich	762024
Tris(3-hydroxypropyltriazolylmethyl)amine (THPTA)	Sigma-Aldrich	762342-100MG
HEPES	Sigma-Aldrich	H3375-100G
NAD, [^{32}P]	Perkin Elmer	BLU023X250UC
GTP, [a-^{32}P]	Perkin Elmer	BLU506H250UC
NAD^+	Sigma-Aldrich	NAD100-RO
HiScribe T7 In vitro Transcription Kit	NEW ENGLAND BioLabs	E2050
iTaq™ Universal SYBR® Green supermix	BioRad	172-5121
Dynabeads™ MyOne™ Streptavidin T1	Thermo Fisher Scientific	65601
complete EDTA-free Protease Inhibitor Cocktail	Roche (Sigma-Aldrich)	11873580001
SYPRO Ruby Protein Gel Stain	BioRad	1703126
3-acrylamidophenylboronic acid	Boron Molecular	BM1195
Cytiva (Formerly GE Healthcare Life Sciences) RPN1210B	Fisher Scientific	45-000-763
Ammonium acetate	Sigma	A-1542
Magnesium chloride	Fisher Scientific	BP214-500
Sodium chloride	Fisher Scientific	BP358-10
Zinc chloride	Sigma	746355
Urea	Fisher Scientific	BP169-10
L-Ascorbic Acid	Sigma	A0278
IPEGAL	Sigma	I-3021
Glycogen	ThermoFisher Scientific	AM9510
Random Primers	Promega	C1181
Oligo(dT)$_{15}$ Primer	Promega	C1101
Mitotracker Deep Red FM	Cell Signaling Technology	#8778

Software and Algorithms

ImageJ		https://imagej.nih.gov/ij/
PRISM-GraphPad	GraphPad	https://www.graphpad.com/scientific-software/prism/
ChemDraw	Perkin Elmer	https://www.perkinelmer.com/category/chemdraw
ImageQuant	GE Healthcare	TL 5.0

Others

PEI-cellulose TLC plates	Sigma-Aldrich	Z122882-25EA
Molecular Dynamics Phosphor Imager	GE Healthcare	Storm 860
Novex™ WedgeWell™ 4 to 20%, Tris-Glycine, 1.0 mm, Mini Protein Gel, 12-well	Thermo Fisher Scientific	XP04202BOX
NAD/NADH Quantitation Kit	Sigma-Aldrich	MAK037-1KT
