# Supplementary material for: Xrn1 is a deNADding enzyme modulating mitochondrial NAD-capped RNA
Source: Nat Commun. 2022 Feb 16;13:889. doi: 10.1038/s41467-022-28555-7 (PMC8850482; doi:10.1038/s41467-022-28555-7)
Supplement: Supplementary file 2 — Description of Additional Supplementary Files [file 41467_2022_28555_MOESM2_ESM.pdf]

Description of Additional Supplementary Files

File Name: Supplementary Data 1
Description: Mass spectrometry identification of proteins selectively bound to the NAD-capped RNA
