# Supplementary material for: Xrn1 is a deNADding enzyme modulating mitochondrial NAD-capped RNA
Source: Nat Commun. 2022 Feb 16;13:889. doi: 10.1038/s41467-022-28555-7 (PMC8850482; doi:10.1038/s41467-022-28555-7)
Supplement: Supplementary file 4 — Reporting Summary [file 41467_2022_28555_MOESM4_ESM.pdf]

nature research

Corresponding author(s): Megerditch Kiledjian

Last updated by author(s): Dec 16, 2021

Reporting Summary

Nature Research wishes to improve the reproducibility of the work that we publish. This form provides structure for consistency and transparency in reporting. For further information on Nature Research policies, see Authors & Referees and the Editorial Policy Checklist.

Statistics

For all statistical analyses, confirm that the following items are present in the figure legend, table legend, main text, or Methods section.

n/a	Confirmed	
☐	☒	The exact sample size (n) for each experimental group/condition, given as a discrete number and unit of measurement
☐	☒	A statement on whether measurements were taken from distinct samples or whether the same sample was measured repeatedly
☐	☒	The statistical test(s) used AND whether they are one- or two-sided<br>*Only common tests should be described solely by name; describe more complex techniques in the Methods section.*
☒	☐	A description of all covariates tested
☒	☐	A description of any assumptions or corrections, such as tests of normality and adjustment for multiple comparisons
☐	☒	A full description of the statistical parameters including central tendency (e.g. means) or other basic estimates (e.g. regression coefficient) AND variation (e.g. standard deviation) or associated estimates of uncertainty (e.g. confidence intervals)
☐	☒	For null hypothesis testing, the test statistic (e.g. F, t, r) with confidence intervals, effect sizes, degrees of freedom and P value noted<br>*Give P values as exact values whenever suitable.*
☒	☐	For Bayesian analysis, information on the choice of priors and Markov chain Monte Carlo settings
☒	☐	For hierarchical and complex designs, identification of the appropriate level for tests and full reporting of outcomes
☒	☐	Estimates of effect sizes (e.g. Cohen's d, Pearson's r), indicating how they were calculated

Our web collection on statistics for biologists contains articles on many of the points above.

Software and code

Policy information about availability of computer code

Data collection	The peak list of the LC-MSMS generated by Thermo Proteome Discoverer (v. 2.1) was searched against the uniprot human fasta database and a database composed of common lab contaminants (CRAP) using in house version of X!Tandem (GPM Fury) (Craig, R. & Beavis, R. C. TANDEM: matching proteins with tandem mass spectra. Bioinformatics 20, 1466-1467, doi:10.1093/bioinformatics/bth092 (2004)).<br>ImageJ software with StackReg plugin was used to correct for XY drift in the images generated for STORM analysis. Images reconstruction, chromatic aberration correction and drift correction was done using ThunderSTORM (Ovesny, M., Krizek, P., Borkovec, J., Svindrych, Z. & Hagen, G. M. ThunderSTORM: a comprehensive ImageJ plug-in for PALM and STORM data analysis and super-resolution imaging. Bioinformatics 30, 2389-2390, doi:10.1093/bioinformatics/btu202 (2014).)<br>GraphPad Prism(V8.20) and Adobe Illustrator and Photoshop (2022) were also used.
Data analysis	Mass spectrometry data was acquired using a data-dependent acquisition procedure with a cyclic series of a full scan with resolution of 120,000 followed by MS/MS (HCD, relative collision energy 27%) of the 20 most intense ions and a dynamic exclusion duration of 20 sec. The peak list of the LC-MSMS were generated by Thermo Proteome Discoverer (v. 2.1).<br>For STORM images, an Olympus 60X 1.3 N.A. UPlanSApo objective was used to acquire 512 X 512-pixel images using an Andor Zyla 4.2 sCMOS camera at 25 frames/second.

For manuscripts utilizing custom algorithms or software that are central to the research but not yet described in published literature, software must be made available to editors/reviewers. We strongly encourage code deposition in a community repository (e.g. GitHub). See the Nature Research guidelines for submitting code & software for further information.

Data

Policy information about availability of data

All manuscripts must include a data availability statement. This statement should provide the following information, where applicable:

- Accession codes, unique identifiers, or web links for publicly available datasets
- A list of figures that have associated raw data
- A description of any restrictions on data availability

Mass Spectrometry data has been deposited at MassIVE with Accession Number: MassIVE MSV000087605 and is publicly available.

Field-specific reporting

Please select the one below that is the best fit for your research. If you are not sure, read the appropriate sections before making your selection.

☒ Life sciences ☐ Behavioural & social sciences ☐ Ecological, evolutionary & environmental sciences

For a reference copy of the document with all sections, see nature.com/documents/nr-reporting-summary-flat.pdf

Life sciences study design

All studies must disclose on these points even when the disclosure is negative.

Sample size	The sample size was chosen from past knowledge on the good sample size to ensure adequate power. Sample size are indicated in figure legends.
Data exclusions	No data exclusions
Replication	Each experiment was performed with at least three independent biological replicates. All experimental results were derived from all experiments.
Randomization	No randomization was performed due to low sample number and the lack of its influence on the experimental design and output.
Blinding	Experiments required previous empirical knowledge for interpretation.

Reporting for specific materials, systems and methods

We require information from authors about some types of materials, experimental systems and methods used in many studies. Here, indicate whether each material, system or method listed is relevant to your study. If you are not sure if a list item applies to your research, read the appropriate section before selecting a response.

Materials & experimental systems

n/a	Involved in the study	
☐	☒	Antibodies
☒	☐	Eukaryotic cell lines
☒	☐	Palaeontology
☒	☐	Animals and other organisms
☒	☐	Human research participants
☒	☐	Clinical data

Methods

n/a	Involved in the study	
☒	☐	ChIP-seq
☒	☐	Flow cytometry
☒	☐	MRI-based neuroimaging

Antibodies

Antibodies used	Commercially available anti-Step tag, -PGK and -MTCO2 antibodies were used.<br>Strep Tag Monoclonal Antibody {GT517) (MAS-17282) from lnvitrogen<br>Anti-MTCO2 (anti-COX2) antibody [4B12A5] (ab110271) from Abca<br>Anti-Pgkl ((PAS-2861) from lnvitrogen<br>Rabbit anti-RFP {600-406-379 from Rockland<br>Mouse anti-GFP {8Hll, 12E6 and Gl) from Developmental Studies Hybridoma Bank<br>Goat anti mouse F (ab)2 Atto 488 {41051-lMG-F from Sigma<br>Goat anti rabbit Alexa Fluor 647 (# A27040) from Thermo Fisher<br>Dilutions used for all the antibodies are included the methods section.
Validation	Validations are carried out by the manufacturers.<br>Strep Tag Monoclonal Antibody {GT517) (MAS-17282) from lnvitrogen:<br>Western blot analysis of Streptavidin using A) 30 µg 293T whole cell lysate and B) 30 µg whole cell lysate of strep-tagged protein

expressing 293T cells. Samples were loaded onto a 12% SDS-PAGE gel and probed with a Streptavidin monoclonal antibody (Product # MA5-17282) at a dilution of 1:5000.

Anti-MTCO2 (anti-COX2) antibody [4B12A5] (ab110271) from Abca: 1/1000 - 1/10000. Predicted molecular weight: 26 kDa.

Anti-Pgkl ((PAS-28612) from lnvitrogen
Predicted reactivity: Mouse (97%), Rat (97%), Zebrafish (87%), Xenopus laevis (84%), Pig (96%), Chicken (88%), Sheep (96%), Rhesus Monkey (100%), Chimpanzee (100%), Bovine (96%).

Goat anti rabbit Alexa Fluor 647 (# A27040) from Thermo Fisher
Immunofluorescence analysis of Goat anti-Rabbit IgG (H+L) Secondary Antibody Alexa Fluor® 647 conjugate was performed using HeLa cells stained with alpha Tubulin Rabbit Polyclonal Antibody (Product # PA5-16891)

Rabbit anti-RFP {600-406-379 from Rockland
This product was prepared from monospecific antiserum by imunoaffinity chromatography using Red Fluorescent Protein (Discosoma) coupled to agarose beads followed by solid phase adsorption(s) to remove any unwanted reactivities. Expect reactivity against RFP and its variants: mCherry, tdTomato, mBanana, mOrange, mPlum, mOrange and mStrawberry. Assay by immunoelectrophoresis resulted in a single precipitin arc against anti-biotin, anti-Rabbit Serum, and purified and partially purified Red Fluorescent Protein (Discosoma). No reaction was observed against Human, Mouse or Rat serum proteins. ELISA was used to confirm specificity at less than 0.1% of target signal.

Goat anti mouse F (ab)2 Atto 488 is from Hypermol Cat #2112

ATTO-labeled F(ab) fragments belong to the new generation of fluorescent antibodies, characterized by their exceptional fluorescence intensity and superior photostability. Anti-Mouse IgG F(ab) ATTO488 is specifically suited for high and super resolution microscopy, due to the high photostability and strong fluorescence quantum yield.

Conjugates of F(ab) fragments are preferable to whole antibody conjugates:

- for cellular structures which are sterically difficult to access;
- prevention of interactions with Fc receptor-bearing membranes due to the absence of the Fc region in Fab fragments;

Mouse anti-GFP {(DSHB Cat# DSHB-GFP-8H11, RRID:AB_2617423)
Previosuly validated (Generating a battery of monoclonal antibodies against native green fluorescent protein for immunostaining, FACS, IP, and ChIP using a unique adjuvant.
Soll DR
Monoclonal antibodies in immunodiagnosis and immunotherapy 33.2 (2014 Apr): 80-8.)
